# MicroRNA-Mediated Epigenetic Regulation of Rheumatoid Arthritis Susceptibility and Pathogenesis

**DOI:** 10.3389/fimmu.2022.838884

**Published:** 2022-03-24

**Authors:** Cen Chang, Lingxia Xu, Runrun Zhang, Yehua Jin, Ping Jiang, Kai Wei, Linshuai Xu, Yiming Shi, Jianan Zhao, Momiao Xiong, Shicheng Guo, Dongyi He

**Affiliations:** ^1^ Guanghua Clinical Medical College, Shanghai University of Traditional Chinese Medicine, Shanghai, China; ^2^ Department of Rheumatology, Guanghua Hospital Affiliated to Shanghai University of Traditional Chinese Medicine, Shanghai, China; ^3^ The Second Affiliated Hospital of Shandong University of Traditional Chinese Medicine, Jinan, China; ^4^ Department of Biostatistics and Data Science, School of Public Health, University of Texas Health Science Center, Houston, TX, United States; ^5^ Center for Precision Medicine Research, Marshfield Clinic Research Institute, Marshfield, WI, United States; ^6^ Department of Medical Genetics, School of Medicine and Public Health, University of Wisconsin-Madison, Madison, WI, United States; ^7^ Arthritis Institute of Integrated Traditional and Western Medicine, Shanghai Chinese Medicine Research Institute, Shanghai, China

**Keywords:** rheumatoid arthritis, microRNA, susceptibility, pathogenesis, epigenetics

## Abstract

MicroRNAs (miRNAs) play crucial roles in regulating the transcriptome and development of rheumatoid arthritis (RA). Currently, a comprehensive map illustrating how miRNAs regulate transcripts, pathways, immune system differentiation, and their interactions with terminal cells such as fibroblast-like synoviocytes (FLS), immune-cells, osteoblasts, and osteoclasts are still laking. In this review, we summarize the roles of miRNAs in the susceptibility, pathogenesis, diagnosis, therapeutic intervention, and prognosis of RA. Numerous miRNAs are abnormally expressed in cells involved in RA and regulate target genes and pathways, including NF-κB, Fas-FasL, JAK-STAT, and mTOR pathways. We outline how functional genetic variants of *miR-499* and *miR-146a* partly explain susceptibility to RA. By regulating gene expression, miRNAs affect T cell differentiation into diverse cell types, including Th17 and Treg cells, thus constituting promising gene therapy targets to modulate the immune system in RA. We summarize the diagnostic and prognostic potential of blood-circulating and cell-free miRNAs, highlighting the opportunity to combine these miRNAs with antibodies to cyclic citrullinated peptide (ACCP) to allow accurate diagnosis and prognosis, particularly for seronegative patients. Furthermore, we review the evidence implicating miRNAs as promising biomarkers of efficiency and response of, and resistance to, disease-modifying anti-rheumatic drugs and immunotherapy. Finally, we discuss the autotherapeutic effect of miRNA intervention as a step toward the development of miRNA-based anti-RA drugs. Collectively, the current evidence supports miRNAs as interesting targets to better understand the pathogenetic mechanisms of RA and design more efficient therapeutic interventions.

## Introduction

Rheumatoid arthritis (RA) is an autoimmune disease characterized by chronic joint inflammation and structural damage, accompanied by extra-articular manifestations such as rheumatoid nodules, interstitial pneumonia, vasculitis, and systemic complications. RA is typically progressive and insidious, with an incidence rate of 0.5–1% in Europe and North America (1). However, the precise mechanisms underlying the pathogenesis, disease activity, and severity of RA as well as the causes of different response to treatment are not fully understood. In view of current therapy strategies and treatment frames, early accurate diagnosis, effective and personalized treatment, and precision medicine have become increasingly urgent for patients with RA. A comprehensive understanding of RA is required from the perspectives of both genetics (2) [human leukocyte antigen (HLA) and non-HLA variants] and epigenetics [DNA methylation (3–5), microRNA (6), long non-coding RNA (7, 8), and histone modifications (9)].

MicroRNAs (miRNAs) are small endogenous non-coding RNAs with a length of around 22 nucleotides, and are involved in the post-transcriptional regulation of gene expression. In recent years, accumulating studies have demonstrated that miRNAs play a key role in various cancers (10–14) and autoimmune diseases, including RA, systemic lupus erythematosus (15, 16), Sjögren’s syndrome (17), and systemic sclerosis (18). In this review, we systematically summarize recent advances in understanding the role of miRNAs in RA. Emphasize the important role of miRNA in RA susceptibility, pathogenesis, and efficacy evaluation. Provide evidence supporting precision medicine research in RA.

## Genetic Variations in miRNAs Explains Susceptibility of RA

Genome-wide association studies have identified >100 genetic factors for RA. However, these genetic variants only explain < 40% of the overall heritability of RA, and thus most of the heritability has not been explained, suggesting the need for more studies using different approaches and populations to identify the missing causes. Association studies of miRNA loci can reveal RA-associated functional or causal variants within different populations, such as Chinese (19, 20), Egyptian (21–23), Polish (24), Mexican (25), and Iranian (26) subjects. Gene expression and genetic polymorphisms of *miR-146a* and *miR-499* showed diagnostic potential for RA (23). Consistently, the polymorphism rs3027898 in *IRAK1*, the target gene of *miR-146a*, is linked to RA in the Greek population (27). In contrast, *miR-146a* rs2431697 is associated with RA susceptibility in Chinese population (28). The rs3746444 (20q11.22, A>G) polymorphism of *miR-499*, which is encoded by the intron of *MYH7B*, is significantly linked to RA risk, disease activity, and methotrexate (MTX) toxicity. Interestingly, the AA genotype shows higher disease activity and MTX toxicity than the AG/GG genotypes (29). The AA and AG genotypes in the miRNA binding site rs3135500 of *NOD2* are significantly associated with the risk of RA, with rs3135500 (A allele) showing a significant relationship with increased erythrocyte sedimentation rates (ESR) and C-reactive protein (CRP) concentrations (30). However, some studies showed inconsistent results in Polish (24), Mexican (25), and Chinese (19, 20, 31, 32) populations, suggesting that genetic polymorphisms of miR-146a and miR-499 are not significantly associated with RA susceptibility. For example, *miR-499* rs3746444 A/G are not significantly associated with RA in Mexican people (25). *miR-146a* rs2910164 (20, 32) and *miR-499* rs3746444 (20, 31) do not significantly correlate with RA in Chinese people. However, in addition to race, factors of individual heterogeneity and sample size should also be considered while evaluating inconsistent results. Moreover, the sample size of the above studies was not large. We recently demonstrated that meta-analysis could identify more significant single-nucleotide polymorphisms in a large sample size, and found that the interaction between HLA alleles and miRNA single-nucleotide polymorphisms (such as rs5997893 in *miR-3928* and rs4947332 in *HLA-DRB1*) should be considered to explain susceptibility (33). In summary, genetic variations in miRNAs can help to explain the susceptibility to RA.

## Regulatory Roles of miRNAs in Cells and Their Secretions Involved in RA Pathogenesis

Fibroblast-like synoviocytes (FLS) and immune cells are the main cell types involved in the pathogenesis of RA. These cells can secrete exosomes and other substances to affect the occurrence and development of RA. Current researches have mainly focused on understanding miRNA-mediated transcriptional regulation of *FAF1* (34), *TNF-α* (35), *STAT1* (36), *STAT3* (37), and *mTOR* (38, 39). miRNAs regulate inflammation, immune response, proliferation, and differentiation. Meanwhile, miRNA influence the micro-environment within synovial joints by targeting target genes and their related pathways, including Fas-FasL (34) and the NF-κB (40, 41) pathways. In this section, we summarize the regulatory roles of miRNAs in the main RA-associated cell entities, focusing on FLS, immune cells, and exosomes to highlight the importance of miRNAs in the pathogenesis of RA.

### Effects of miRNAs on FLS in RA

FLS in RA (RA-FLS) are key regulators of inflammation and bone destruction in RA. The aberrantly expression of miRNAs in RA-FLS play an important role in the pathogenesis of RA. For example, *miR-625* is down-regulated in RA-FLS, which negatively impacts the expression of *CTSC*, *KLF8*, and *EBF3*. In contrast, *miR-551b* is up-regulated in RA-FLS, inhibiting the expression of *ITGBL1* (42).

Dysregulation of miRNAs in RA-FLS affects biological functions such as cell proliferation, invasion, migration and apoptosis. Up-regulation of *miR-145* affects all biological functions of RA-FLS by targeting *SEMA3A* (43). The expression of *miR-29c-3p* and *miR-132-3p* are decreased while *miR-31-5p* was increased in RA- FLS, and their dysregulation are associated with proliferation, invasion and migration of RA-FLS. Down-regulated *miR-29c-3p* promoted proliferation, invasion and migration of RA-FLS through up-regulation of *COLA1* expression. Interestingly, up-regulated *miR-31-5p* and down-regulated *miR-132-3p* inhibited the proliferation, invasion and migration of RA-FLS by negatively regulating *WASF3* and *RB1*, respectively, suggested that *miR-31-5p* and *miR-132-3p* are protective factors in RA (44). Down-regulation of *miR-199a-3p* (45)*, miR-449* (46)*, miR-431-5p* (47), and up-regulation of *miR-483-3p* (48) can promote the proliferation and suppressed apoptosis by targeting *RB1*, *HDAC1*, *XIAP*, *IGF-1*, respectively. *miR-124a* is down-regulated and targets *CDK2 and MCP-1* which only enhanced the proliferation of RA-FLS (49).

Dysregulation of miRNAs in RA-FLS can also affects the level of inflammation. Down-regulation of *miR-126* (35), *miR-23* (50) and up-regulation of *miR-143* (43) can increase the release of inflammatory factors such as TNF-α, IL-1β, IL-6 through up-regulation of *IL-23R*, *CXCL12* and down-regulation of *IGFBP5* and thus affect the course of RA. What’s more, some miRNAs not only affects the level of inflammation, but also associated with biological functions of RA-FLS. For example, down-regulation of *miR-137* (51) and *miR-23a-5p* (52) targeting LSD and TLR4 promotes proliferation, invasion, migration and inhibits apoptosis of RA-FLS and inhibits the release of inflammatory factors IL-1β and IL-6. Down-regulation of *miR-29a* (37) and *miR-27a-3p* (53) are associated with proliferation, apoptosis, and promoting secretion of TNF-α, IL-1, IL-6 and IL-8. Then, down-regulation of *miR-22* (54), *miR-124* (55) and *miR-34a-5p* (56) can enhanced proliferation of RA-FLS and the level of IL-6.

Additionally, the biological functions of RA-FLS and the level of inflammation are correlated with matrix metalloproteinases (MMPs) (57). Down-regulation of *miR-203* (41, 42) and *miR-147b-3p* (58) increased the expression of *MMP-1*, *MMP3* and *MMP9*, respectively, which in turn enhanced the expression of some inflammatory factors, such as IL-6 and TNF-α. Down-regulation of *miR-27a* (59) can contribute to *MMPs gene* expression by targeting the IL-17 pathways, thereby affecting the proliferation and invasion of RA-FLS. Conversely, up-regulated *miR-155* could be a protective factor by inhibiting proliferation and invasion while attenuating the expression of *MMP3* and *IKBKE* (60, 61).

In addition, dysregulation of miRNAs in RA-FLS affects joint bone erosion through the release of inflammatory cytokines, chemokines and MMPs, which may be an option for RA treatment (62, 63). For example, up-regulated *miR-145-5p* (40) and down-regulated *miR-17-5p* (64) affect bone and cartilage destruction through the release of IL-1β, IL-6, IL-8, *MMP-1*, *MMP-3* and *MMP-9*. *In vitro*, overexpression of *miR-221-3p* inhibits osteoblast differentiation (65). Instead, *miR-218* overexpression promotes osteogenic differentiation of RA-FLS by suppressing the Roundabout-1/Dickkopf-1 axis (66). *miR-20a* (67) and *miR-21* (68) are targets of the TLR4/p38 and JAK/STAT3 signaling pathways respectively, affecting the proliferation and osteogenic differentiation of RA-FLS.

**Figure 1:**
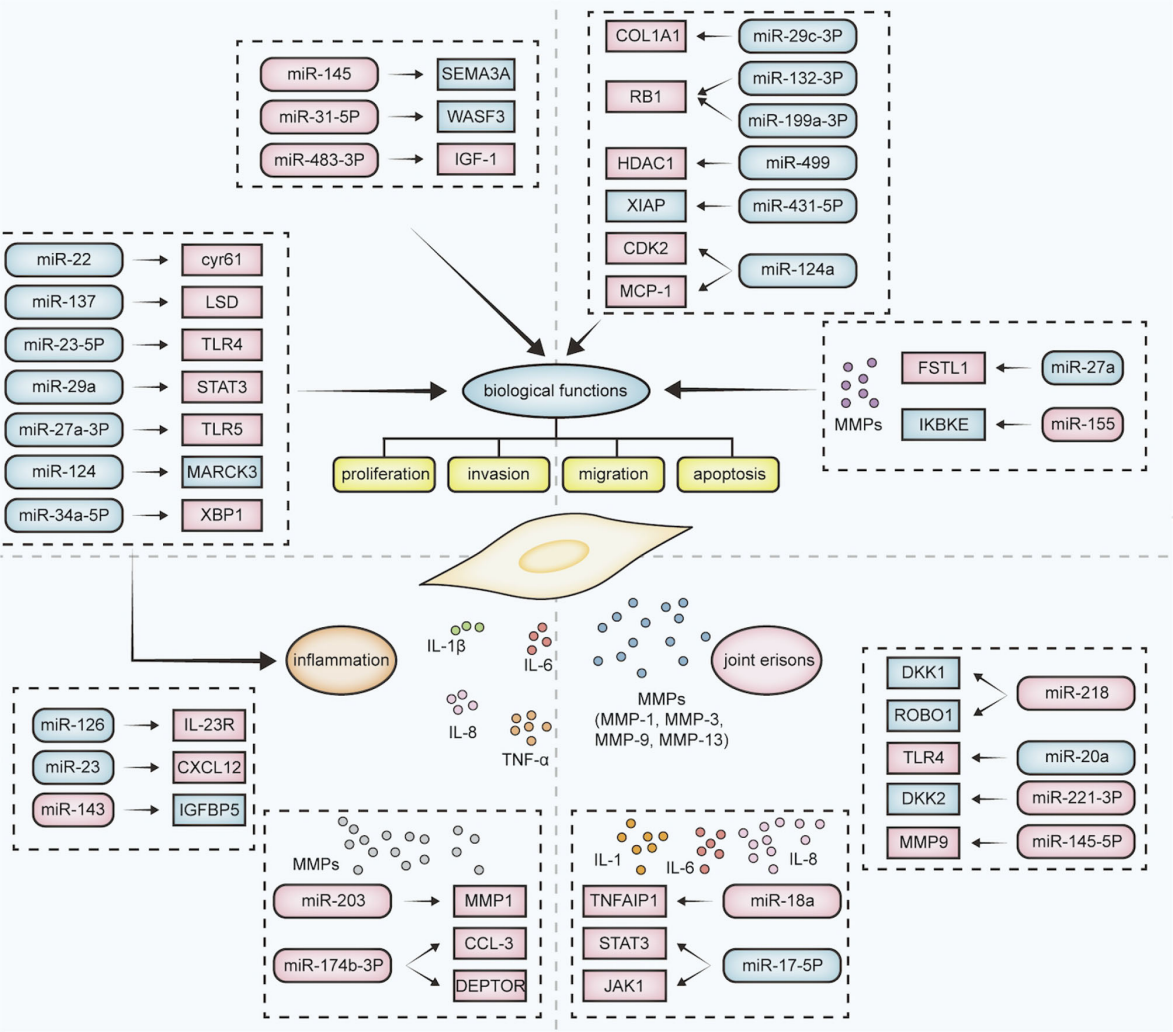
Effects of dysregulation of the miRNA-mRNA network on RA-FLS. The dysregulation of miRNAs and their target mRNAs in RA-FLS affects the biological function (such as proliferation, invasion, migration, and apoptosis), inflammatory levels, and joint bone destruction. Inflammatory levels are mainly related to the release of inflammatory factors such as IL-8, IL-6, and joint bone destruction is mainly related to the release of MMPs. Dysregulation of different miRNA-mRNA combinations affects different processes in RA-FLS. Rounded rectangles represent miRNAs; rectangles represent target mRNAs; pink represents upregulation; blue represents downregulation.

In summary, studies of miRNAs in RA-FLS have improved the understanding of the pathogenesis of RA. miRNAs are widely involved in the functions of FLS, and therefore are promising targets for drug development (**Figure 1**).

### Effects of miRNAs on Immune Cells in RA

miRNAs have recently emerged as key regulators of the immune system, being involved in lymphocyte selection and proliferation, in T(reg) cells differentiation. In peripheral blood mononuclear cells (PBMCs), decreased expression of *miR-671* and *miR-7* may correlate with the expression of *CDR1* and *mTOR* (38). And *miR-29b* enhances the anti-apoptotic effect by inhibiting the high-mobility group box-containing protein 1 (HBP1) (69).

In T cells sub-population derived from PBMCs, *miR-99b-5p* down-regulates *mTOR* and *RASSF4*, thereby inhibiting T cell apoptosis and promoting T cell proliferation and inflammatory response (39). Besides, *miR-146a, miR-26, miR-let-7a, miR-146b, miR-150, miR-155* are increased and *miR-363, miR-498* are decreased in the CD4^+^ T cell sub-type in PBMCs, Amon*g these m*iRNAs, *miR-146a* may affect the apoptosis of T cell and RA progression by targeting *IL-17* and Fas associated factor 1 (*FAF1*) (34, 70). Interestingly, *miR-233* is highly expressed only in naive CD4+ lymphocytes but not in T(h)-17 cells, suggesting the importance to investigate the impact of miRNA on the pathogenesis of RA at the single-cell level (71).

There are numerous of miRNAs are also associated with other T cells sub-types, such as Treg cells and Th17 cells. The balance of Th17/Treg cells plays a crucial role in RA. IL-17 released by Th17 up-regulates the expression of *RANKL* on synovial fibroblasts stimulating the production of inflammatory cytokines such as TNF-α, IL-1, and IL-6 (72). Decreased levels of *miR-20a* (73) and *miR-21* (74, 75) exacerbate the RA process by stimulating the *NLRP3* inflammasome pathway and increasing *STAT3* expression, respectively, while decreasing *STAT5* expression, all of which are associated with the imbalance of Th17/Treg cells. Although *miR-210*-mediated negative regulation of *HIF-1* also affects the dynamic equilibrium of Th17/Treg cells. Regrettably, the levels of *miR-210* between RA and healthy controls have no significant difference (76). Interestingly, the expression of *miR-146a* is decreased in Treg cells during high RA activity, leading to a proinflammatory phenotype in these cells caused by concomitant up-regulation of its target *STAT1* (36). For instance, *miR-21* and *miR-155* are related to the memory phenotype, and *miR-92a* relative to the naïve phenotype (77).

Besides, in macrophages, binding of *miR-6089* and *lncRNA-HIX003209* enhances the expression of *TLR4* and exacerbates inflammation *via* the TLR4/NF-κB pathway (78). Up-regulation of *miR-33* induces the expression of *NLRP3* and 73 (79). Overall, miRNAs cooperate with other non-coding RNAs to alter the DNA methylation and/or expression of their targets, thus regulating innate and adaptive immune cells differentiation and apoptosis, ultimately influencing the inflammatory and autoimmune response in RA (**Table 1**).

**Table 1:** Effects of microRNAs on immune cells in rheumatoid arthritis.

miRNA	miRNA trends	Targets	Targets trends	Location	Functions	Reference
miR-671	↓	CDR1	↑	PBMC	/	(40)
miR-7	↓	mTOR	↑			
miR-29b	↑	HBP1	↑			(73)
miR-99b-5p	↑	mTOR	↑	T cell	inhibiting T cell apoptosis, promoting T cell proliferation, inflammatory response	(41)
		RASSF4				
miR-146a	↑	FAF1	↑		inhibiting apoptosis of T cell and RA progression	(35)
		IL-17	↑			(74)
miR-26	↑	/	/		/	(35, 74)
miR-let-7a	↑					
miR-146b	↑					
miR-150	↑					
miR-155	↑					
miR-363	↓					
miR-498	↓					
miR-233	↑					(75)
miR-20a	↓	NLRP3	↑	Treg/Th17	the imbalance of Th17/Treg cells	(77)
miR-21	↓	STAT3	↑			(78, 79)
		STAT5	↓			
miR-210	↓	HIF-1	↑			(80)
miR-146a	↓	STAT1	↑	Treg cells	/	(38)
miR-6089	↓	TLR4	↑	macrophages	exacerbates inflammation *via* the TLR4/NF-κB pathway	(81)
miR-33	↑	NLRP3	↑		/	(82)
		caspase-1				

### Effects of miRNAs on Cell Secretions in RA

Exosomes are secreted from cells and contain signal molecules such as miRNA, protein, and DNA, which have biological functions. Exosomal miRNA derived from bone marrow-derived mesenchymal stem cells has been shown to be closely related to the occurrence and development of RA. Among these exosomes, MSCs-drived *miR-124a* over-expression exosomes inhibit the proliferation and migration and promote the apoptosis of RA-FLS (80). Over-expression of *miR-23b* (83) and *miR-34a* (81) can inhibit the differentiation of Th17 cells, by reducing IL-17 secretion and targeting the cyclin I/ATM/ATR/p53 signaling pathway, respectively. Up-regulated of *miR-21* (82) which targets *TET1*, reduce RA inflammation. Macrophage-derived exosomes *miR-506-3p* (84) and *miR-103a* (85) regulate the progression of RA by inhibiting the RANKL/NFATc1 signaling pathway and activating the JAK/STAT3 signaling pathway. *miR-132* secreted by aryl hydrocarbon receptor activation Th17 in extracellular vesicles acts as a pro-inflammatory mediator to reduce the production of *COX2*, to increase the production of osteoclast (86). In addition, cell-derived small extracellular vesicles of *miR-574-5p* induces osteoclast differentiation by targeting *TLR 7/8* (87), whereas *miR-150-5p* exosomes alleviates RA-FLS proliferation and angiogenesis and reduces RA joint destruction by targeting *MMP14* and *VEGF* (88). Based on these results, miRNAs play an important role in the pathogenesis of RA and may represent promising outcome biomarkers and novel drug targets to decrease disease severity.

## Blood and Serum-Circulating miRNAs Provide Novel Opportunities for Precision Medicine of RA

### miRNAs as Potential Biomarkers for Early Prevention and Precision Diagnosis

Emerging evidence indicates the potential of blood-circulating miRNAs associated with RA as biomarkers for early prevention. The levels of *miR-371b*, *miR-483*, and *miR-642b* are significantly up-regulated, whereas *miR-25* and *miR-378d* are down-regulated in PBMCs in individuals who eventually develop RA from early undifferentiated arthritis (89). Additionally, *miR-22* (90), *miR-361-5p* (91), and *miR-223-3p* (91) are significantly up-regulated in high-risk or CCP-positive populations. All these miRNAs may therefore be useful biomarkers for the early diagnosis of RA. Expression of *miR-103a-3p* is significantly increased in autoantibody-positive, symptomatic first-degree relatives and patients with RA, suggesting it as a potential biomarker for predicting imminent disease in individuals at risk for developing RA (92). Additionally, higher level of *miR-99b-5p* is found in the plasma of patients with early RA who progress to bone erosion after 12 months, indicating that *miR-99b-5* can be monitored for bone erosion surveillance in RA patients (93).

In addition to playing a role in the early prevention of RA, the expression of some miRNAs can aid in improving the accuracy of RA diagnosis (94). The expression of *miR-146a* and *miR-155* are significantly increased in RA PBMCs and whole blood (95). The levels of *miR-24* and *miR-125a* are significantly higher in the serum of patients with RA regardless of the CCP status (96). Interestingly, analysis of *miR-24-3p*, *miR-26a-5p*, and *miR-125a-5p* levels in combination are a better diagnostic tool for RA, even though these miRNAs are not related to disease activity (97). Furthermore, *miR-122-3p*, *miR-3925-3p*, *miR-342-3p*, and *miR-4764-5p* show differential expression not only between healthy individuals and RA patients, but also between patients with RA and patients with osteoarthritis, systemic lupus erythematosus, or Graves, which show great potential as biomarkers to distinguish RA patients from HC or other diseases (98). The serum levels of *miR-146a* (99, 100), *miR-22-3p* (101), *miR-5571-3p* (102), and *miR-135b-5p* (102) are significantly higher in RA patients than in healthy controls and osteoarthritis patients. Other differentially expressed miRNAs in patients with RA serum include *miR-4634*, *miR-181d*, *miR-3926*, *miR-9-5p*, *miR-219-2-3p6*, *miR-221*, *miR-222*, *miR-532*, *miR-106a*, and *miR-98* highlighting their potential as RA-specific diagnostic markers (98, 103). Nevertheless, the above miRNAs should be selected as biomarkers with caution. Their sensitivity and specificity need to be taken into consideration because they were only compared with patients with osteoarthritis (OA) or healthy control, and rarely analyzed with patients with other inflammatory autoimmune diseases, such as ankylosing spondylitis. In addition, it should be determined if the miRNA as diagnostic markers are expressed differently in patients before the onset of clinical symptoms. The above studies were conducted after patients were confirmed with RA diagnosis. Thus, more robust sample studies are needed to validate these markers in early RA.

### miRNAs as Potential Biomarkers for Disease Activity and Treatment Response

The expression of *miR-451* in T cells is significantly increased, which is positively correlated with the levels of disease activity score 28 (DAS-28), ESR, and serum IL-6 in RA (77). The level of *miR-146a* is positively correlated with the level of ESR and DAS-28 (99), whereas *miR-5571-3p* (102) correlates with the level of ESR and CRP, and *miR-135b-5p* only correlate with CRP (102). These miRNAs may therefore be suitable markers of disease activity in patients with RA. Increased serum *miR-194-5p* level is associated with disease recurrence (104). Concentration of circulating *miR-23b*, which positively correlates with ESR, CRP, and DAS-28, is significantly up-regulated after appropriate treatment, indicating that *miR-23b* is a dual marker for disease activity and prognosis (105). Similarly, *miR-96-5p, miR-134-5p, miR-140-3p, miR-627-5p, miR-224, miR-760, miR-483-5p, miR-378, and miR-375* are not only diagnostic markers for RA, but also mirror disease activity (106, 107). However, these studies are still descriptive. Therefore, the underlying pathophysiology needs to be validated using other techniques.

Common and widely used anti-rheumatic drugs include cDMARDs (MTX, sulfasalazine, and hydroxychloroquine), bDMARDs (TNF-α inhibitors, rituximab, and tocilizumab), tsDMARDs (tofacitinib, barretinib, and filgotinib). Several studies have explored the relationship between serum miRNA levels and drug response. Evidence shows that high serum levels of miR-10 in patients with RA is correlated with good response to MTX (108). After 3 months of adalimumab/MTX combined treatment, the level of *miR-27a-3p* significantly decreased and clinical symptoms significantly improved (109). The reduced serum level of *miR-5196* is positively correlated with the delta DAS28 after anti-TNF-α therapy (110). The level of *miR-146a* is increased in RA patients who respond well to anti-TNF therapy and, interestingly, can be considered as predictors of the response to anti-TNFα therapy together with CRP (24, 111, 112). In contrast, the serum levels of *miR-23* and *miR-223* are increased in patients with RA who respond well to anti-TNF-α/DMARD combination therapy, but correlate negatively with the response to anti-TNF drugs (111). High serum level of *miR-125b* is an indicator for good clinical response to rituximab therapy (113). Notably, *miR-432-5p* is significantly down-regulated in RA patients who are responsive to tofacitinib therapy but up-regulated in patients showing RA relapse (104). In RA, treatment with rituximab increases the levels of *miR-16-5p* and *miR-23a-3p* in the peripheral blood (114). The expression of *miR-550b-2-5p*, *miR-4797-5p*, *miR-6509-5p*, *miR-378g*, *miR-4720-5p*, *miR-374b-5p*, and *miR-185-3p* are different between individuals who show good *vs* poor responses to treatment with tripterygium glycosides (115, 116). Finally, the expression of *miR-124a* in FLS is increased following geniposide treatment; however, the relevance of this finding has not been assessed in clinical response studies (117).

In addition to DMARD treatment, alternative and complementary medicine preparations and mesenchymal stem cell treatments are also used in clinical practice. The auto-therapeutic effect of miRNAs has been demonstrated in mouse models of RA-FLS and autoimmune arthritis. For example, *miR-449a* mimics also inhibit the proliferation, migration, and IL-6 production of RA-FLS by regulating *HMGB1* and *YY1* expression (118). In the rat model with collagen-induced arthritis, *miR-708-5p* mimic improved pathological changes by inhibiting inflammatory cell infiltration, synovial hyperplasia, and cartilage destruction (119). An *miR-126* agonist inhibits the expression of IL-23R, TNF-α, and IFN-γ in FLS (35). Furthermore, *miR-26b-5p*, *miR-487b-3p*, and *miR-495-3p* are significantly up-regulated in responders to adipose-derived mesenchymal stem cell treatment (120).

In summary, the changes of circulation miRNA in RA provide a promising opportunity for standard treatment, as well as indicate disease activity and predict RA outcomes.

## miRNAs Research in RA: Remaining Challenges and Future Opportunities

In conclusion, miRNAs play multiple roles in the development of RA, from susceptibility to pathogenesis. Blood and serum-circulating miRNAs have been explored as important biomarkers for early diagnosis, prognosis, and drug response prediction. Furthermore, miRNAs have been proposed for autotherapeutic approaches and as novel drug targets for the treatment of RA. Genetic variants in specific miRNAs can increase or decrease the risk and disease activity of RA in various racial. Meanwhile they are associated with methotrexate toxicity and responses to other treatments. Moreover, changes in miRNAs in various cells are related to the pathogenesis of RA, such as the proliferation and differentiation of immune cells, proliferation and apoptosis of synovial cells, and synovial inflammation and cartilage destruction. Research has remarkably progressed towards the development of miRNAs as biomarkers in the diagnosis, prognosis, disease activity, and response to therapeutic drugs with RA, providing a direction for early diagnosis and accurate treatment of RA, to achieve better treatment efficiency and precision medicine. Numerous miRNAs have been shown to act as therapeutic targets in RA-FLS and collagen-induced arthritis rat models. Furthermore, miRNAs show potential for identifying the subtypes of RA. For example, the levels of *miR-7* and *miR-214-5p* are significantly increased in the serum of patients with RA associated-interstitial lung disease (121), and *miR-9-5p* targets the REST/miR-132 pathway to protect Schwann cells from inflammatory damage in RA-induced peripheral neuropathy (122). Although we have reached exciting milestones in the research on the multiple roles of miRNA in RA, further studies should be performed to translate this knowledge for clinical applications and resolve the current inconsistent results among different studies employing different methods or populations. For example, studies of *miR-99*, *miR-143*, and *miR-197* as landmark miRNAs for predicting the response to anti-TNF-α therapy have failed to yield consistent results (123). Finally, future development of miRNA-based baseline RA polygenetic risk score models, particularly in conjunction with HLA, is needed. miRNA-based early diagnosis, prognosis, and drug response prediction models can be applied in the clinic. With the identification of additional miRNAs-based drug targets in clinical research, miRNA-based autotherapeutic treatments may show more promising results.

## Author Contributions

SG and DH conceived the content. CC and LXX wrote the manuscript. RZ, YJ, PJ, KW, and MX edited the manuscript. YS, JZ and LSX checked the manuscript information. LXX wrote the manuscript and LSX checked the manuscript information.

## Funding

This work was funded by the National Natural Science Funds of China (82074234 and 82004166); Shanghai Chinese Medicine Development Office, National Administration of Traditional Chinese Medicine, Regional Chinese Medicine (Specialist) Diagnosis and Treatment Center Construction Project-Rheumatology; State Administration of Traditional Chinese Medicine, National TCM Evidence-Based Medicine Research and Construction Project, Basic TCM Evidence-Based Capacity Development Program; Shanghai Municipal Health Commission, East China Region based Chinese and Western Medicine Joint Disease Specialist Alliance.

## Conflict of Interest

The authors declare that the research was conducted in the absence of any commercial or financial relationships that could be construed as a potential conflict of interest.

## Publisher’s Note

All claims expressed in this article are solely those of the authors and do not necessarily represent those of their affiliated organizations, or those of the publisher, the editors and the reviewers. Any product that may be evaluated in this article, or claim that may be made by its manufacturer, is not guaranteed or endorsed by the publisher.
